# Generation of tissue-engineered small intestine using embryonic stem cell-derived human intestinal organoids

**DOI:** 10.1242/bio.013235

**Published:** 2015-10-12

**Authors:** Stacy R. Finkbeiner, Jennifer J. Freeman, Minna M. Wieck, Wael El-Nachef, Christopher H. Altheim, Yu-Hwai Tsai, Sha Huang, Rachel Dyal, Eric S. White, Tracy C. Grikscheit, Daniel H. Teitelbaum, Jason R. Spence

**Affiliations:** 1 Department of Internal Medicine, Division of Gastroenterology, University of Michigan Medical School, Ann Arbor, MI 48109, USA; 2 Center for Organogenesis, University of Michigan Medical School, Ann Arbor, MI 48109, USA; 3 Department of Surgery, Section of Pediatric Surgery, University of Michigan Medical School, Ann Arbor, MI 48109, USA; 4 Developmental Biology and Regenerative Medicine Program, Saban Research Institute, Children's Hospital, Los Angeles, CA, USA; 5 Department of Internal Medicine, Section of Pulmonary and Critical Care, University of Michigan Medical School, Ann Arbor, MI 48109, USA; 6 Department of Cell and Developmental Biology, University of Michigan Medical School, Ann Arbor, MI 48109, USA

**Keywords:** Human intestinal organoids, Tissue-engineered intestine, Matrix, Scaffold

## Abstract

Short bowel syndrome (SBS) is characterized by poor nutrient absorption due to a deficit of healthy intestine. Current treatment practices rely on providing supportive medical therapy with parenteral nutrition; while life saving, such interventions are not curative and are still associated with significant co-morbidities. As approaches to lengthen remaining intestinal tissue have been met with only limited success and intestinal transplants have poor survival outcomes, new approaches to treating SBS are necessary. Human intestine derived from embryonic stem cells (hESCs) or induced pluripotent stem cells (iPSCs), called human intestinal organoids (HIOs), have the potential to offer a personalized and scalable source of intestine for regenerative therapies. However, given that HIOs are small three-dimensional structures grown *in vitro*, methods to generate usable HIO-derived constructs are needed. We investigated the ability of hESCs or HIOs to populate acellular porcine intestinal matrices and artificial polyglycolic/poly L lactic acid (PGA/PLLA) scaffolds, and examined the ability of matrix/scaffolds to thrive when transplanted *in vivo*. Our results demonstrate that the acellular matrix alone is not sufficient to instruct hESC differentiation towards an endodermal or intestinal fate. We observed that while HIOs reseed acellular porcine matrices *in vitro*, the HIO-reseeded matrices do not thrive when transplanted *in vivo*. In contrast, HIO-seeded PGA/PLLA scaffolds thrive *in vivo* and develop into tissue that looks nearly identical to adult human intestinal tissue. Our results suggest that HIO-seeded PGA/PLLA scaffolds are a promising avenue for developing the mucosal component of tissue engineered human small intestine, which need to be explored further to develop them into fully functional tissue.

## Abstract (precis)

**Summary:** HIO-seeded PGA/PLLA scaffolds thrive *in vivo* and develop into tissue that looks nearly identical to adult human intestinal tissue. These scaffolds appear to be suitable for further tissue engineering approaches to develop functional intestine.

## INTRODUCTION

Short bowel syndrome (SBS) is characterized by poor nutrient absorption due to a deficit of healthy intestine after surgical removal of diseased tissue and is associated with a loss of greater than 50% of normal intestinal length (Spencer et al., 2005; Sukhotnik et al., 2005; Wales and Christison-Lagay, 2010). SBS often affects neonates and children and has mortality rates up to 10–30% (Hess et al., 2011; Spencer et al., 2005; Vanderhoof and Langnas, 1997). Within the pediatric patient population, necrotizing enterocolitis is becoming the most common disease leading to SBS (Modi et al., 2008; Spencer et al., 2008; Wales et al., 2005). In addition to the high mortality and morbidity, there are high, long-lasting costs of care for SBS patients (Spencer et al., 2008). Current treatment practices focus on a multidisciplinary approach to providing supportive medical therapy with supplemental or total parenteral nutrition (TPN) (Sulkowski and Minneci, 2014). While such measures have generally improved survival and parenteral weaning rates (Hess et al., 2011; Modi et al., 2008), there are still complications associated with TPN including metabolic complications (hyperglycemia, hypophosphatemia), liver failure and catheter-related morbidity and sepsis (ChrisAnderson et al., 1996; Sulkowski and Minneci, 2014). Therefore, treating diseases that lead to SBS is a major challenge in pediatric gastroenterology (Goday, 2009).

The length of remaining healthy tissue is a key determinant of patient outcomes in SBS. After surgery, the intestine adapts and increases in circumference and length in an attempt to compensate for the loss of tissue (Dekaney et al., 2007; Garrison et al., 2009; McDuffie et al., 2011; Seetharam and Rodrigues, 2011). Surgical lengthening procedures have been explored to exploit this phenomenon but result in far less than a 2-fold increase in length (Chang et al., 2006; Khalil et al., 2012; Oliveira et al., 2012; Scott et al., 2015). Intestinal transplants are an alternate approach; however, graft rejection rates near 60% within five years post surgery (Seetharam and Rodrigues, 2011). Therefore, new approaches are to treat SBS needed. We have described a system that allows us to efficiently differentiate human embryonic stem cells (hESCs) and induced pluripotent stem cells (iPSCs) into three-dimensional intestinal tissue, called human intestinal organoids (HIOs) *in vitro* (Finkbeiner and Spence, 2013; McCracken et al., 2011; Spence et al., 2010), and have demonstrated that HIOs develop adult architectural and molecular features when placed into an *in vivo* environment such as a mouse kidney capsule (Finkbeiner et al., 2015; Watson et al., 2014). Since induced pluripotent stem cells can be generated from patient cells through cellular reprogramming (Takahashi et al., 2007), iPSCs are a valuable source of patient-specific tissue that could be used for tissue engineering approaches aimed at generating autologous small intestine for transplantation.

While HIOs may be a viable approach to treat SBS, how to scale small HIO constructs into viable intestine remains a challenge. Here, we explored two distinct approaches to create scaffolds for tissue engineering the small intestine: (1) decellularized porcine intestinal ECM scaffolds and (2) porous polyglycolic/poly L lactic acid (PGA/PLLA) scaffolds (Barthel et al., 2012; Grant et al., 2015; Levin et al., 2013; Sala et al., 2011; Wulkersdorfer et al., 2011). We reasoned that either of these approaches, if successful, would be scalable in order to generate a tissue engineered small intestine (TESI) construct suitable for transplantation. Moreover, PGA/PLLA scaffolds have been successfully used to generate TESI constructs from human cells and donor tissue (Costello et al., 2014; Grant et al., 2015; Levin et al., 2013).

Here, we demonstrate that decellularized porcine intestine is a tractable substrate for reseeding with HIOs *in vitro*, but proves less promising for use *in vivo* or for providing lineage-specific differentiation cues for human pluripotent stem cells. In contrast, use of a PGA/PLLA scaffolds supports HIO growth *in vivo* and results in the development of a tissue that is strikingly similar to the native adult human intestine, with characteristic architectural features and cell types with the correct spatial organization of intestinal cells relative to adult human small intestine. However, HIO-seeded scaffolds lack important elements required for full functionality such as an enteric nervous system, which is involved in motility. We demonstrate as proof-of-principle that additional cellular inputs are able to provide neuronal components, which integrate into the scaffold adjacent to HIO-derived epithelium. Taken together, our results suggest that HIOs can be successfully used to generate TESI constructs, and that PGA/PLLA scaffolds are suitable for further tissue engineering approaches to develop functional intestine.

## RESULTS

**Fig. 1.**
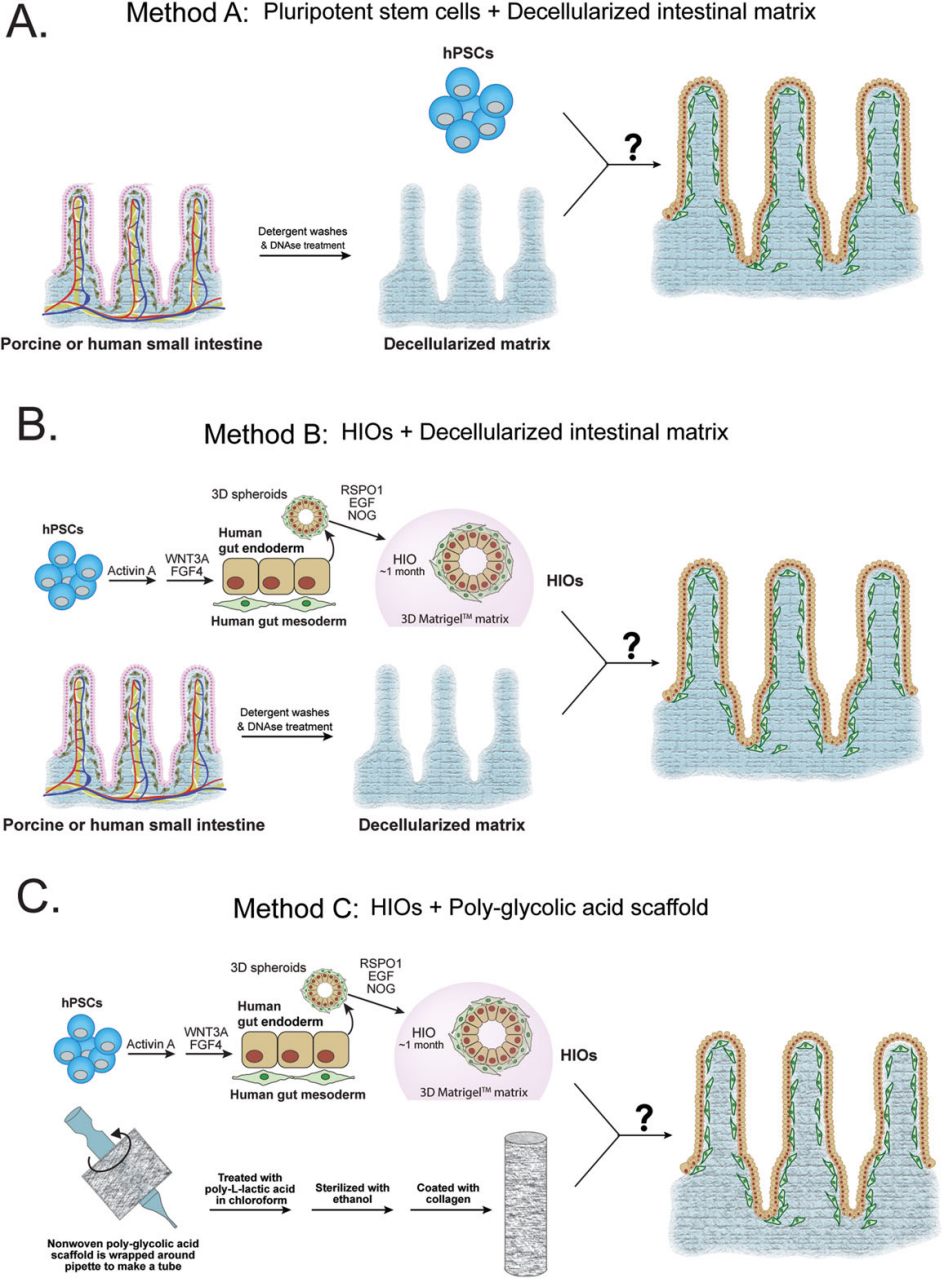
**Schematic of approaches to generating bioengineered intestine.** (A) Porcine or human small intestine were harvested and processed through a series of detergent washes followed by treatment with sodium deoxycholate and DNase in order to remove cells and remaining nuclei and DNA content. The acellular scaffolds were then reseeded with human pluripotent stem cells and analyzed for the ability of the cells to efficiently reseed the matrix and whether the matrix could push them towards and intestinal identity while being culture *in vitro*. (B) Porcine or human small intestine were harvested and processed through a series of detergent washes followed by treatment with sodium deoxycholate and DNase in order to remove cells and remaining nuclei and DNA content. The acellular matrix was then reseeded with HIOs and analyzed for the ability of the cells to efficiently reseed the matrix and whether different intestinal cell populations were present. They were analyzed after both *in vitro* culturing and after implantation into an immunocompromised mouse. (C) Nonwoven polyglycolic acid scaffolds were wrapped around a glass pipette and treated with poly-L-lactic acid and chloroform to secure the tubular structure. They were then sterilized and treated with collagen. HIOs were seeded onto the outside of the scaffold and then immediately implanted into an immunocompromised mouse and analyzed for the ability of HIOs to reseed the scaffold and for the presence of different intestinal cell types.

**Fig. 2.**
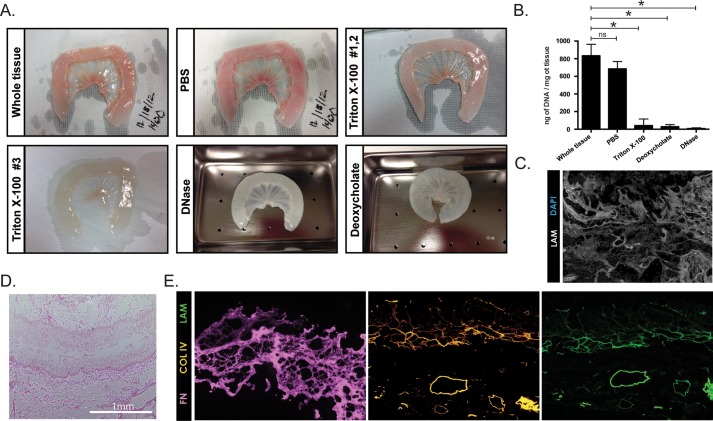
**Decellularization of porcine small intestine.** (A) Porcine small intestine is shown starting from whole tissue and then after subsequent stages of the decellularization protocol resulting in the final acellular matrix that has a translucent appearance. (B) Small pieces of porcine intestine were collected at various stages of the decellularization protocol to measure DNA content confirming that the protocol successfully removes existing DNA as there is a significant reduction after the detergent washes and then the DNA content reaches zero by the final step of DNAse treatment. *N*=3 for each group. *P<0.05 based on an unpaired *t*-test using Welch's correction, error bars represent s.e.m. (C) Porcine matrix was DAPI stained to confirm a lack of nuclei and co-stained with laminin to outline the matrix. (D) Hematoxylin and eosin staining of a cross section of the matrix shows the preservation of the matrix structure with finger-like projections into the lumen, which presumably represent the inner extracellular matrix cores of what were previously villi. Scale bar: 1 mm. (E) Serial cross sections of matrix were stained for extracellular matrix proteins to further confirm the integrity of the acellular matrix. Collagens and laminin are still present even after decellularization suggesting the decellularization process does not remove all extracellular matrix proteins.

Successful seeding of a scaffold with HIOs or precursor cells is the first step in developing a transplantable tissue-engineered intestine. A necessary complement to this first step is determining which cells are capable of reseeding the scaffold and how those cells behave on the scaffold over time. Tissue-engineered intestine suitable for transplantation will need to exhibit the characteristics of normal intestine by containing all of the appropriate differentiated intestinal cell types including enterocytes, goblet cells, Paneth cells, intestinal stem cells (ISCs), enteroendocrine cells and intestinal mesenchymal cells, while lacking other lineages that are not present in the intestine. We took a multi-pronged approach to generating TESI utilizing native and engineered scaffolds and starting with both embryonic stem cells (hESCs) and human intestinal organoids (HIOs) (Finkbeiner and Spence, 2013; McCracken et al., 2011; Spence et al., 2010). Native scaffolds were prepared by decellularizing both porcine and human small intestine following a protocol that has been previously used to prepare acellular lung scaffolds (Booth et al., 2012) (Figs 1, 2, Fig. S1). Following decellularization, acellular intestinal matrix was reseeded with two different cell sources: hESCs (Fig. 1A) and HIOs (Fig. 1B). Collagen substrates have been shown to support maintenance and expansion of epithelial cells and an artificial scaffold made of polyglycolic/poly L lactic acid (PGA/PLLA) has previously been shown to be a successful substrate for generating TESI from minced human small intestine (Grant et al., 2015; Jabaji et al., 2014, 2013; Levin et al., 2013). Therefore, we also tested this approach by seeding HIOs onto PGA/PLLA scaffolds (Fig. 1C).

### Decellularization of intestinal matrix

Segments of porcine and human small intestine were subjected to a series of detergent washes followed by DNase treatment in order to remove all cellular components from the extracellular matrix (ECM) (Fig. 2A). Acellular scaffolds were also acid-treated for sterilization. Human intestine was challenging to decellularize due to copious visceral fat associated with the mesentery (Fig. S1). Thus, while we were able to successfully decellularize the human intestine, we chose to focus on the use of porcine intestine, as it is also a more readily available source of matrix. DNA content was measured at various stages during the decellularization process and efficient removal of cells and DNA from the intestinal tissues was confirmed (Fig. 2B). Intestinal matrix was sectioned and examined using nuclear DAPI stain to visually confirm the removal of all nuclear material from host cells (Fig. 2C). To ensure that our decellularization process does not substantially alter the architecture of the intestinal matrix, we also examined histological sections of matrix using hematoxylin and eosin. The gross architecture appears to be in tact given that villus projections are still visible (Fig. 2D). Furthermore, extracellular matrix proteins laminin, collagen II and IV, which are expressed in the intestine (Barnes et al., 2011; Simon-Assmann et al., 1995), are detectable after decellularization (Fig. 2E).

### Acellular intestinal matrix does not induce differentiation of human pluripotent stem cells

**Fig. 3.**
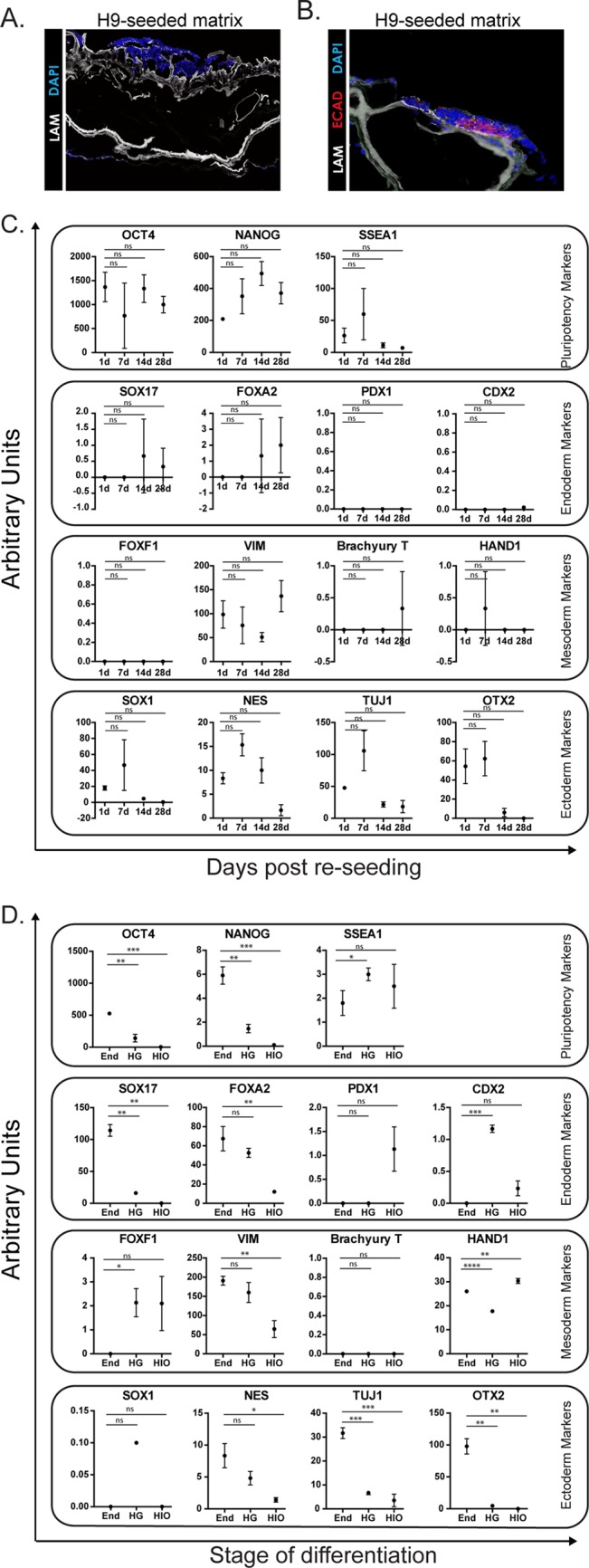
**Acellular porcine matrices seeded with pluripotent stem cells.** (A) H9 human pluripotent stem cells (hPSCs) are able to reseed acellular porcine matrix but do not penetrate into the matrix very well as examined after 4 weeks post seeding. (B) Some stem cells that were seeded onto the matrix expressed Ecad when analyzed 4 weeks post seeding suggesting that at least a portion of the hPSCs had begun to differentiate. (C) hPSC-seeded porcine matrices were analyzed by qRT-PCR at different time point to examine the expression of stem cell, endoderm, mesoderm, and ectoderm markers. From this analysis, it does not appear that porcine matrix drives hPSCs in any specific direction of differentiation. (D) Cells were examined at different stages during the development of HIOs to provide a reference for part C. As expected, pluripotency markers are low and endodermal markers go up during the development process. Note scale differences of the *y*-axes between C and D for some genes. *N*=3 for each sample type. *P<0.05 based on an unpaired *t*-test using Welch's correction, error bars represent s.e.m.

We wanted to explore the possibility that native acellular intestinal extracellular matrix could provide instructive cues to promote differentiation of human embryonic stem cells (hESCs) into intestinal tissue and thereby present an option for reseeding these scaffolds. To test this possibility, we seeded H9 hESCs onto small (∼3 mm×3 mm) full-thickness sections of acellular porcine matrix and monitored their growth *in vitro* for up to 4 weeks with daily changes of mTeSR™1 stem cell media. Gross analysis based on media utilization suggested that the stem cells adhered to the matrix and were able to be maintained for up to 4 weeks in culture. hESC-seeded matrices were collected and examined by histology at weekly intervals. Sections of hESC-seeded matrix showed clumps of adherent cells present on the surface of the matrix (Fig. 3A,B). Cells did not appear to migrate/infiltrate into the matrix. Moreover, cell clumps appeared to be a heterogeneous population after 4 weeks with only some of the cells expressing the epithelial cell marker E-cadherin (ECAD) (Fig. 3B). The observation that seeded hESCs were heterogeneous suggested that some of them had differentiated.

To examine the identity and potential differentiation state of hESCs seeded onto matrix, RNA was collected at weekly intervals for 4 weeks post-seeding. hESC-seeded matrices were examined for markers of pluripotency (*OCT4*, *NANOG*, *SSEA1*) as well as markers of the three germ-layers that the hESCs could become: endoderm (*SOX17*, *FOXA2*, *PDX1*, *CDX2*), mesoderm (*FOXF1*, *VIM*, *T*, *HAND1*), and ectoderm (*SOX1*, *NES*, *TUJ1*, *OTX2*) (Fig. 3C) and compared to the expression of these genes in conditions where stem cells were directed to become endoderm, followed by hindgut (HG), followed by HIOs (Fig. 3D, note differences in *y*-axes). Collectively, our results demonstrate that hESCs seeded onto acellular porcine intestinal matrix did not undergo lineage-specific differentiation, suggesting that the matrix alone does not provide instructive cues for differentiation. We observed that stem cell genes *OCT4* and *NANOG* are highly expressed on hESC-seeded matrices throughout the 4 weeks in culture though *SSEA1* is more variable in expression and appears to decrease over time (Fig. 3C, top panel). This was in contrast to directed differentiation, which showed reduced expression of stem cell genes at different stages (Fig. 3D, top panel). Endodermal markers *SOX17* and *FOXA2* which are highly expressed in endoderm were not highly expressed at any time point in the hESC-seeded matrices and there was no detection of *PDX1* or the intestinal transcription factor *CDX2* that are both highly expressed in HIOs, suggesting that the matrix itself cannot induce differentiation of hESCs into endodermal lineages or intestine (compare second panel Fig. 3C versus Fig 3D). In hESC-seeded matrices, mesodermal markers were generally low with the exception of *VIM*, whereas directed differentiation cultures showed expression of different mesenchymal markers, consistent with the fact that these cultures possess small portions of mesoderm due to inefficient differentiation (Spence et al., 2010) (compare third panel Fig. 3C versus Fig 3D). Lastly, hESC cultures showed variable expression of ectoderm markers whereas directed differentiation cultures had reduced expression of these markers at each stage indicating lineage-specific differentiation (compare third panel Fig. 3C versus Fig 3D). Collectively, these data show that hESCs seeded onto acellular porcine intestinal matrix were not driven down any specific lineage pathway. In addition, it did not appear that hESCs re-seed the matrix with high efficiency. Together, this data supports the notion that hESCs are not a good cell source for TESI using an acellular scaffold.

### HIOs efficiently reseed acellular intestinal matrix *in vitro*

**Fig. 4.**
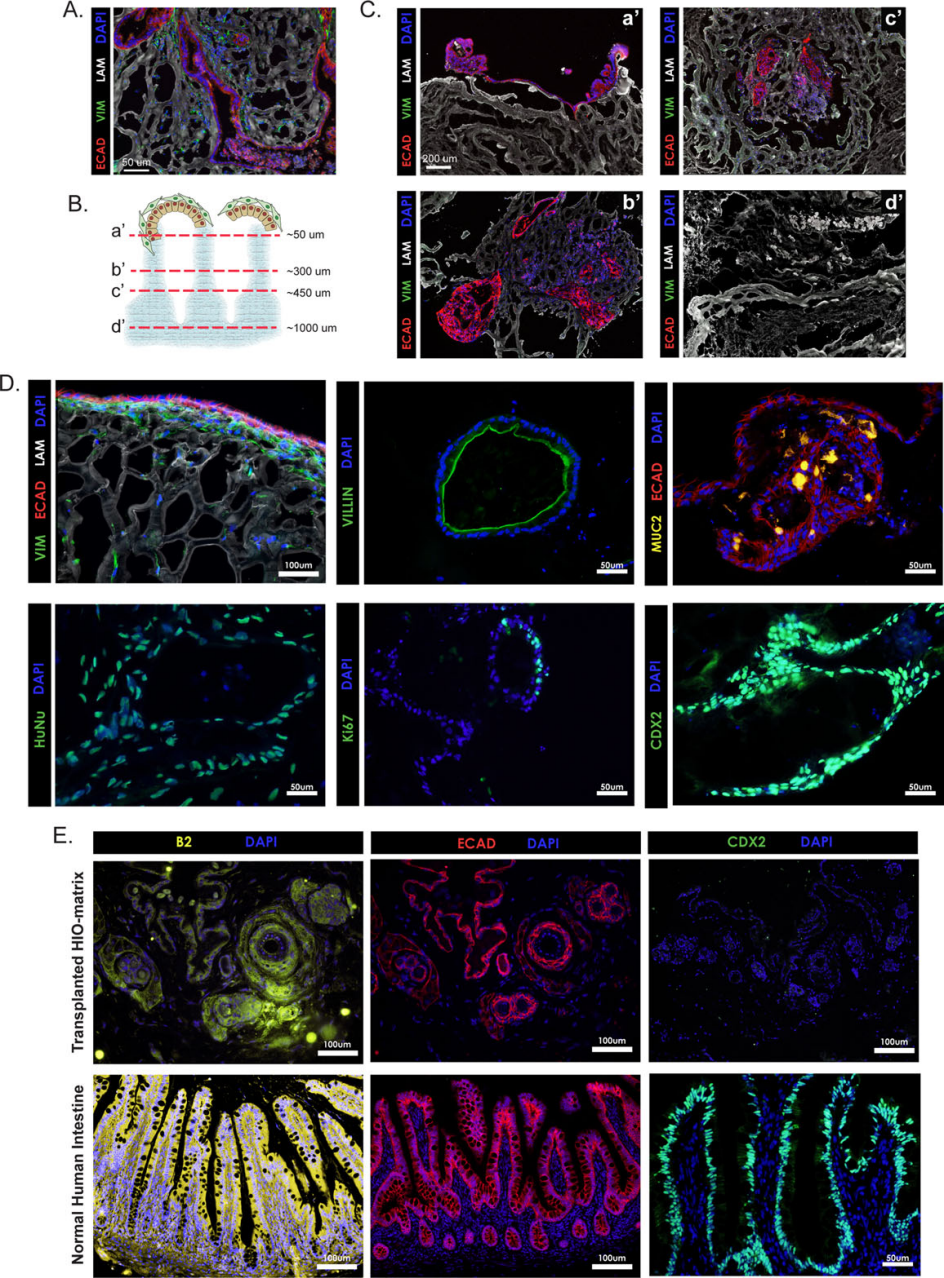
**Acellular porcine matrices seeded with HIOs.** (A) HIOs are able to reseed acellular matrices and grow *in vitro* as shown by staining for epithelial (ECAD+) and mesenchymal (VIM+) cells at 4 weeks post seeding. (B,C) Serial sections progressing from the luminal to serosal surface of a matrix show that HIOs are able to migrate into the matrix and do not solely reside on the surface of the matrix. (D) HIO-seeded matrices show appropriate spatial orientation in which epithelial cells line the luminal surface of the matrix and mesenchymal cells are oriented underneath (upper, right). Human nuclear antigen staining confirms that the cells present on the matrices are of human origin and therefore derived from HIOs (lower, right). VILLIN and MUC2 staining show that HIO-seeded matrices support at least some differentiated intestinal cell types, enterocytes and goblet cells respectively (upper, middle and right). Ki67 staining shows proliferating cells present on the matrix and CDX2 staining further confirms that the cells are of an intestinal identity (lower, middle and right). (E) HIO-reseeded matrices were implanted into immunocompromised mice and analyzed 14 weeks later. β2 microglobulin staining revealed that some matrices retained human cells (top left). ECAD staining showed that these cells were epithelial (top middle) but the lack of CDX2 staining demonstrates they were not intestinal cells (top right). Images from human intestine are shown for comparison (bottom row). Scale bars: 50 μm in A, D, (except upper left), E (lower right); 100 μm in E (except lower right), D (upper left); 200 μm in C.

Following a step-wise, directed differentiation approach that mimics signaling events occurring during normal intestine development *in vivo* (Fig. 1A), we are able to generate HIOs (Finkbeiner and Spence, 2013; McCracken et al., 2011; Spence et al., 2010). HIOs contain an outer layer of mesenchymal cells that surrounds a complex epithelium. The epithelium contains the major differentiated cell types found in the small intestine, including absorptive cells (enterocytes), secretory cell lineages (Paneth, goblet, enteroendocrine), as well as putative intestinal stem cells. The intestinal identity and presence of both epithelium and mesenchyme suggest HIOs might be an ideal cell source of scalable tissue for reseeding acellular native matrices. Therefore we generated HIOs according to our standard protocol (McCracken et al., 2011). HIOs were cut in half and placed on the luminal (epithelial) side of full thickness acellular matrix and cultured for up to 4 weeks *in vitro*. HIOs attached to the matrices and both epithelial (ECAD+) and mesenchymal (VIM+) cells were present (Fig. 4A). In comparison to the hESCs, which remained on the surface of the matrix, HIOs appeared to infiltrate the matrix. To evaluate the extent of infiltration, serial cross sections were taken through an HIO-seeded matrix and evaluated for the presence of cells (Fig. 4B,C). The thickness of the matrix is ∼1000 μm and cells could be detected down through ∼600 μm. The remaining ∼300–400 μm on the serosal side of the matrix lacked cells.

On reseeded matrices, epithelial cells were often observed along the luminal surface of the matrix with an underlying layer of mesenchymal cells beneath (Fig. 4D, upper left). Staining for human nuclear antigen (HuNu) confirmed that the cells present on the matrix were of human origin and therefore not an artifact of incomplete decellularization (Fig. 4D). HIO-seeded matrices were further examined to determine the status of proliferation and differentiation of the cells. As expected, a heterogeneous population of cells was present on the matrix. Some were proliferating based on Ki67 staining and other expressed markers of differentiated cell types. Enterocytes (VILLIN+) and goblet cells (MUC2+) were both readily detected (Fig. 4D). The intestinal transcription factor CDX2 was also readily detected suggesting the HIO-derived cells seeded onto the matrix retain an intestinal phenotype.

Given the success in reseeding acellular matrices with HIOs and culturing them *in vitro*, we next implanted HIO-reseeded matrices into immunocompromised (NOD-SCID IL2Rgamma null) mice (Ito et al., 2002) in order to evaluate the ability of the TESI construct to engraft and survive *in vivo*. Matrices were reseeded with HIOs and cultured *in vitro* for 2 weeks before being implanted subcutaneously, into the omentum or under the kidney capsule. These regions were chosen for preliminary *in vivo* experiments because they are highly vascularized, and are often used as sites for tissue transplantation (Raghavan et al., 2011). Seven HIO-seeded porcine matrices were implanted into 5 mice. Matrices were retrieved and analyzed 14 weeks after implantation. Only 2/7 matrices contained human cells as confirmed by staining for human specific β2 microglobulin (Fig. 4E). One was implanted subcutaneously and the other into the kidney capsule. The human cells were restricted to single large patches on each matrix. These cells were ECAD+; however, they were not positive for the intestinal transcription factor CDX2 (Fig. 4E), nor were they positive for the intestinal differentiation markers MUC2 or VILLIN (data not shown). From these data it was evident that very few HIO-derived cells persisted on the matrix and those that no longer had an intestinal phenotype. It should also be noted that multiple initial implantation experiments were conducted for shorter lengths of time (2–4 weeks) under various conditions (with and without pumps to infuse the growth factor PDGF), but no human cells were detected on the matrices in these experiments (data not shown). Therefore, although HIO-seeded native matrices thrive in culture they do not survive as *in vivo* implants under the experimental conditions reported here.

### HIOs seeded onto PGA/PLLA scaffolds thrive *in vivo* and develop intestinal architecture

**Fig. 5.**
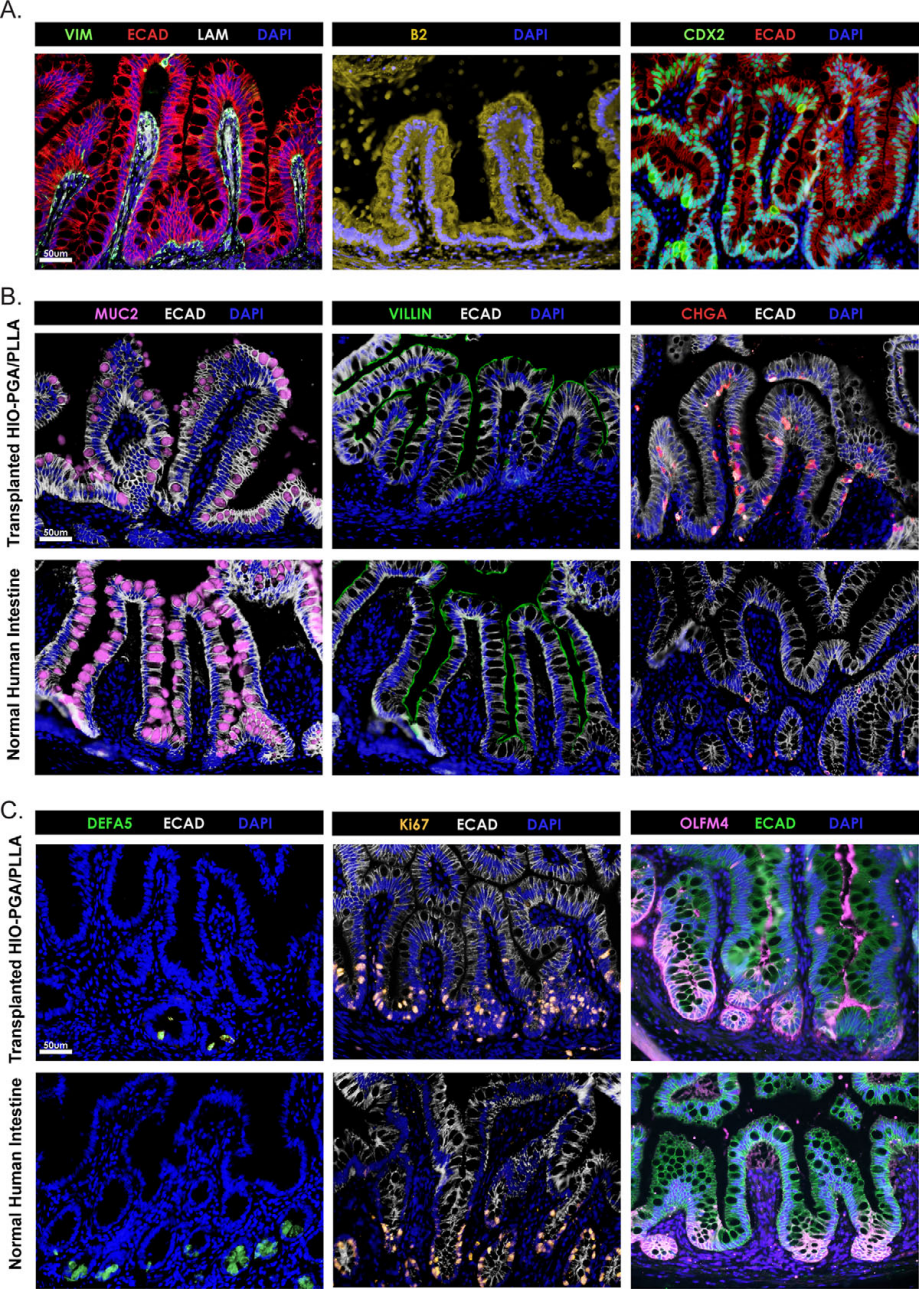
**PGA/PLLA scaffolds seeded with HIOs.** (A) PGA/PLLA scaffolds were seeded with HIOs, implanted into immunocompromised mice, and analyzed 13 weeks later. HIOs successfully reseeded PGA/PLLA scaffolds and developed villus structures (top left). β2 microglobulin (top middle) and CDX2 (top right) staining confirmed the human and intestinal identities of the cells, respectively. (B) Staining confirms that cells found on the HIO-seeded PGA/PLLA scaffolds include goblet cells (MUC2; top left), enterocytes (VILLIN; top middle), and enteroendocrine cells (chromogranin A, ChgA; top right) which are all cells types that normally reside on the villi. Normal human small intestine is shown for comparison (bottom row). (C) HIO-seeded scaffolds develop crypts where Paneth cells (DEFA5; top left) and proliferating cells (Ki67; top middle) including intestinal stem cells (OLFM4; top right) are concentrated. Normal human small intestine is shown for comparison (bottom row). Scale bars: 50 μm.

It has been demonstrated that the use of polymer scaffolds made from PGA/PLLA is promising for generating human TESI when seeded with minced human small intestinal tissue (Levin et al., 2013). Given the limitations of acquiring patient-specific intestinal tissue and the chance of rejection if using heterologous tissue, we wanted to explore the use of HIOs as an alternative source of intestinal cells on PGA/PLLA scaffolds. To test this possibility, we seeded HIOs onto PGA/PLLA scaffolds using previously reported methods (Barthel et al., 2012; Levin et al., 2013; Sala et al., 2011). HIOs were seeded onto PGA/PLLA scaffolds and immediately implanted into NSG mice. Scaffolds were harvested and analyzed after 12 weeks. Unlike with HIO-seeded porcine matrices, which did not dramatically change size during the implantation period, HIO-seeded PGA/PLLA scaffolds started out a similar size as porcine matrices when implanted but were much larger at the time of harvest (Fig. S2). Immunohistochemical analysis of HIO-seeded PGA/PLLA scaffolds revealed efficient seeding of the scaffold resulting in impressive intestinal architecture demonstrated by the presence of epithelial (ECAD) lined villi containing mesenchymal (VIM) positive cores (Fig. 5A versus Fig. 4E, lower panel). β2 microblobulin and CDX2 staining confirm the human and intestinal identity of the cells on the scaffold, respectively (Fig. 5A).

Markers of the major differentiated cells types located in the small intestine were detected on the HIO-seeded scaffolds. These included goblet cells (MUC2), enterocytes (VILLIN), and enteroendocrine cells (CHGA) (Fig. 5B). Furthermore, the HIO-seeded scaffolds also contained crypt domains as evidenced by the presence of mature Paneth cells marked by alpha-defensin 5 (DEFA5) staining, proliferating Ki67+ cells, and intestinal stem cells marked by OLFM4 (Fig. 5C). It should be noted that experiments in which the scaffolds were recovered after only 4 weeks lacked prominent intestinal architecture suggesting that longer transplant times were important to obtain intestinal architecture (data not shown). However, when provided enough time, the HIO-seeded scaffolds developed into tissue that appears nearly indistinguishable from healthy human adult small intestine.

**Fig. 6.**
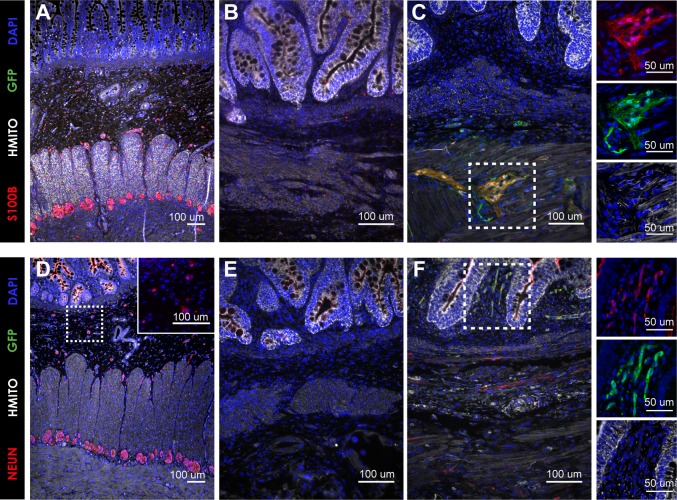
**PGA scaffolds seeded with HIOs and GFP OU.** The first group of PGA scaffolds were seeded with HIOs alone and the second were seeded with HIOs supplemented with OU derived from *actin^GFP^*mice. All constructs were implanted into the omentum of adult NOD/SCID mice and then harvested at 12 weeks. (A) Normal human small intestine contains glia (S100b) in the submucosal and myenteric plexi. (B) Staining tissue engineered intestine from HIO alone demonstrated no S100b+ glia. (C) Supplementation with murine GFP OU resulted in GFP+/hMito−/S100b+ glia predominantly in the myenteric plexus. (D) Normal human small intestine contains neurons (NeuN) in the submucosal and myenteric plexi. (E) Tissue engineered intestine derived from HIO alone demonstrated no NeuN+ structures. (F) Supplementation with GFP OU resulted in GFP+/hMito−/NeuN+ neurons predominantly in the submucosal layer. Scale bars: 100 μm; 50 μm in C and F, insets.

Fully functional tissue engineered intestine will require cellular components other than just the mucosa. For example, normal human small intestine contains glia (S100b+) and neurons (NeuN+) in the submucosal and myenteric plexi (Fig. 6A,D), which are involved in intestinal motility (Bassotti et al., 2007). An additional experiment was carried out to evaluate the presence of neuronal cell types in HIO-seeded PGA/PLLA scaffolds. PGA/PLLA scaffolds seeded with HIOs lacked S100b or NeuN positive cells (Fig. 6B,E). However, supplementation of HIO-seeded PGA/PLLA scaffolds with previously described organoid units (OU) (Barthel et al., 2012; Levin et al., 2013; Sala et al., 2011) prepared from *actin^GFP^* mice resulted in GFP+/S100b+ glia in the myenteric plexus (Fig. 6C) and GFP+/NeuN+ neurons predominantly in the submucosal layer (Fig. 6F). The S100b+ and NeuN+ cells were negative for human mitochondrial staining (hMito) confirming they are derived from the murine organoid units as indicated by the GFP signal. Importantly, GFP+ neuronal elements were adjacent to hMito+ epithelium suggesting that the neuronal elements integrated into the scaffold with the HIO-derived epithelium. This indicates that while transplantation of HIOs in PGA/PLLA scaffolds results in impressive architectural development, they are still lacking neurons, and will need to be paired with additional cellular inputs to completely recapitulate human small intestine.

## DISCUSSION

We pursued multiple approaches to generating tissue engineered human small intestine starting from hESCs or HIOs and either acellular intestinal matrices or artificial polymer scaffolds. We showed that we could successfully decellularize porcine intestine in order to use it as a substrate for seeding HIOs to create tissue engineered small intestine. A perceived advantage of using acellular intestinal matrices is that the matrix itself may retain cues to promote intestinal differentiation or cell survival. However, stem cells seeded onto porcine matrices were not induced to take on an endodermal or intestinal phenotype, suggesting that decellularized matrix prepared via our described protocol lacked the required signals for stem cell differentiation. Porcine matrices could be readily reseeded and maintained *in vitro* with HIOs. However, these reseeded matrices did not perform well once placed in an *in vivo* context as few matrices retained any human cells and the few human cells that did remain were not intestine. It is unclear why this was the case as we have previously shown that HIOs that are implanted on their own into mice will grow and develop crypt-villus architecture (Watson et al., 2014). One possible hypothesis is that the matrix actually blocked infiltration of necessary cells, such as the vasculature, that are required to provide critical support to maintain the HIOs *in vivo*. It is also possible that our method of decellularization removed important growth factors or other cues that could promote cell survival and engraftment *in vivo*. Indeed, in future experiments it is possible that different decellularization methods may prove to enhance *in vivo* engraftment.

Although HIOs can develop intestinal architecture on their own when placed *in vivo*, they grow as small closed spheres, or as small multi-luminal structures, and therefore would ultimately be difficult to connect with existing small intestine, especially when scaling up to generate a construct that could be used to effectively treat a patient. Therefore, a tubular shaped scaffold that has the ability to be reseeded by multiple HIOs alleviates some of these challenges. We demonstrated that HIOs grow quite well *in vivo* on a tube-shaped PGA/PLLA scaffold and the resulting mucosal tissue is nearly indistinguishable from normal adult human small intestine and components of the enteric nervous system can be identified when supplemented with additional cell types. Our results demonstrate a proof-of-principle that HIOs in combination with a PGA/PLLA scaffold offer a promising approach to generating tissue engineered human intestine but that further inputs will be required to generate fully functional tissue. Future work will be required to test the scalability of this approach and to further test the functionality of these bioengineered tissues and ability to connect them with existing intestine.

## MATERIALS AND METHODS

### Culturing of hPSCs and generation of HIOs

H9 human embryonic stem cells (Wicell International Stem Cell Bank, Wicell Research Institute; Madison, WI, USA) were cultured under feeder-free conditions in mTeSR1**^®^** (StemCell Technologies; Vancouver, BC, CA) following standard protocols (Yermen, 2014). HIOs were generated as previously described (McCracken et al., 2011; Spence et al., 2010; Xue et al., 2013). The University of Michigan Human Pluripotent Stem Cell Research Oversight (HPSCRO) committee approved all work using hESCs.

### Decellularization of native intestinal matrices

Human intestine was obtained from deceased donors to the Michigan Gift of Life program. Institutional IRB approval was obtained for use of human tissues (University of Michigan IRB #HUM000105750). All animal experiments (pig, mouse) were approved by the appropriate animal care use committees (PRO00004854, PRO00004296). The decellularization protocol was adapted from a protocol used to decellularize lungs (Booth et al., 2012). Briefly, ∼6–8 inch-long intestinal segments were flushed with PBS hourly for 8 h and then left rocking overnight in PBS. After overnight washing in PBS, intestinal segments were rinsed again in PBS and flushed with 0.1% Triton X-100 (Sigma-Aldrich, St Louis, MO, USA) hourly for 8 h and left rocking in the solution in between flushings. Intestinal segments were then flushed with 2% sodium deoxycholate (Sigma-Aldrich) and left rocking for 4 h. Segments were washed with PBS and flushed with 1 M NaCl (Sigma-Aldrich) for 1 h to lyse nuclei followed by one 4 h treatment with DNase (10,000 units; Life Technologies, Carlsbad, CA, USA) in 1.3 mM MgSO_4_ (Life Technologies) and 2 mM CaCl_2_ (Life Technologies). After DNase treatment, matrices were sterilized in 0.18% peracetic acid and 4.8% ethanol (Sigma-Aldrich) for 30 min, washed three times in sterile PBS, and then stored in sterile PBS containing fungizone (2.5 μg/ml, Life Technologies) and penicillin/streptomycin (100 μ/ml, 100 μg/ml, Life Technologies).

### *In vitro* seeding experiments

See Fig. 1 for a summary of *in vitro* seeding experiments. Decellularized intestines were cut open and divided into ∼3 mm×3 mm-square sections. Each matrix section was then placed into a single well of a 96-well tissue culture treated plate with the mucosal surface facing up to be reseeded with either stem cells or human intestinal organoids (HIOs).

To reseed matrices with stem cells, H9 hESCs (Wicell International Stem Cell Bank, Wicell Research Institute; Madison, WI, USA) growing under feeder-free conditions were treated with dispase (Invitrogen, Carlsbad, CA, USA) and mechanically released from tissue culture dishes using a cell scraper. Colonies were broken into smaller sized aggregates of stem cells as is normally done for splitting of stem cells. Fragmented colonies totaling ∼100×10^4^ stem cells were pipetted directly onto the mucosal surface of each 3 mm×3 mm matrix and allowed to adhere for 30 min before the matrices were completely covered with mTeSR1^®^ (StemCell Technologies, Vancouver, BC, Canada) stem cell media. The following day, matrices were transferred to a fresh 96-well in order to completely remove the matrices from any remaining unattached stem cells. Matrices were then fed on a daily basis with fresh mTeSR1^®^ (StemCell Technologies) and collected for analysis at 1, 2, and 4 weeks post seeding.

To reseed matrices with HIOs, 1–2 month old organoids were removed from matrigel and cut in half using a scalpel. Excess matrigel was also trimmed away from the HIOs. Using forceps, the organoid halves were placed epithelial side face down onto the mucosal surface of the matrices. HIOs used for these experiments were generally all approximately the same size and 5 halves were placed onto each matrix. HIO-seeded matrices were placed in a tissue culture incubator for 1 h without any media to allow the HIOs to attach to the matrix prior to covering them with the standard growth media for HIOs. Media was replaced every 2–3 days as needed and HIO-seeded matrices were collected at 1 day, 1 week, 2 week, and 4 week time points post seeding.

### Implants of native matrices

Immediately before implantation, implants were marked with a 6-0 prolene stitch in the corner to assist with identification at explant. After induction of appropriate anesthesia, mice underwent either a midline or right subcostal incision. Mice with midline incisions had implants placed in the omentum and subcutaneously. Mice with subcostal incisions had implants placed in the renal capsule and subcutaneously. Reseeded scaffolds were left in host for 14 weeks and mice were euthanized immediately prior to removal.

### Generation of PGA/PLLA scaffolds

As previously described (Sala et al., 2011), the scaffold consisted of 2 mm thick nonwoven polyglycolic acid with a porosity of >95% (Concordia Fibers). This polymer was wrapped around a 1.5 mm diameter glass capillary pipette and then treated with 5% poly-L-Lactic acid (Durect Corporation, Cupertino, CA, USA) in chloroform (Sigma-Aldrich), thus allowing it to retain a tubular form. The scaffold was sterilized with 75% ethanol and then coated with 0.4 mg/ml type I collagen (Sigma-Aldrich) at 4°C for 20 min. After rinsing with sterile phosphate buffered saline, the polymer was stored in a desiccator at room temperature to prevent hydrolysis and degradation. On the day of the experiment, 3 mm-wide sections of the tubular polymer were cut prior to being seeded with intestinal organoids.

### Implants of PGA/PLLA scaffolds

Intestinal organoids were removed from matrigel and their normal growth media and were individually transferred by gentle pipetting onto the outer surface of the scaffold, for a total of 20 HIOs per scaffold, with a total of 4 scaffolds. For experiments involving organoid units, organoid units were derived from *actin^GFP^* mice, prepared as previously described (Barthel et al., 2012; Levin et al., 2013; Sala et al., 2011), and mixed with HIOs prior to seeding on each scaffold. Similarly to a previously used implantation protocol (Barthel et al., 2012), each seeded scaffold was surgically implanted within the omentum of a host NOD/SCID gamma mouse (Jackson Laboratories, Bar Harbor, ME, USA) (Ito et al., 2002) irradiated with 350 cGY prior to implantation to prevent immunologic rejection. The omentum was retracted from the mouse peritoneum, wrapped around the seeded scaffold, and sealed by suturing to securely contain the implant. The implant/omentum unit was then reduced back into the peritoneal cavity and the surgical incision was sutured closed. Post-operative treatment included 3 days of 2 ml ibuprofen (100 mg/5 ml; Major Pharmaceuticals, Livonia, MI, USA) and 12 weeks of 2 ml of sulfamethoxazole (200 mg/40 mg/5 ml; Qualitest Pharmaceuticals, Huntsville, AL, USA) per 200 ml autoclaved drinking water. After 12 weeks, host mice were euthanized within a CO_2_ chamber and implants were harvested and fixed in formalin prior to preparation in paraffin for histologic evaluation.

### DNA extraction

Tissues were weighed prior to extraction and then DNA was extracted using a DNeasy Blood and Tissue Kit (Qiagen, Valencia, CA, USA) following the manufacturer's protocol. DNA concentrations were measured using a Nanodrop Instrument and normalized to the starting tissue weight.

### Immunofluorescence

Paraffin sections were stained following standard histology and immunofluorescence protocols as previously described (Dye et al., 2015; Spence et al., 2010). Primary antibodies used are listed in Table S1. Images were collected using an Olympus IX71 epifluorescent microscope (Olympus Corporation, Center Valley, PA, USA). All post acquisition image processing (brightness and contrast) was applied uniformly to all comparable images. Image adjustments were made for clarity and do not obscure, eliminate or misrepresent the original data.

### qRT-PCR

RNA was extracted from re-seeded matrices and HIOs alone using Trizol (Life Technologies) and following the manufacturer's protocol. SuperScript^®^Vilo™ reverse transcriptase (Life Technologies) was used to generate cDNA and qRT-PCR reactions were carried out using QuantiTect Sybr^®^ Green (Qiagen). Reactions were run under the following conditions: 40 cycles of 95°C for 15 s, 55°C for 30 s, and 72°C for 45 s, followed by a melt curve of 95°C for 15 s, 60°C for 1 min and then increasing temperature up to 95°C at 0.5° increments. Primer sequences were provided in the Human qPrimerDepot (http://primerdepot.nci.nih.gov/) and are listed in Table S2.
